# Albumin nano-encapsulation of caffeic acid phenethyl ester and piceatannol potentiated its ability to modulate HIF and NF-kB pathways and improves therapeutic outcome in experimental colitis

**DOI:** 10.1007/s13346-018-00597-9

**Published:** 2018-11-14

**Authors:** Murtaza M. Tambuwala, Mohammed N. Khan, Paul Thompson, Paul A. McCarron

**Affiliations:** 1 SAAD Centre for Pharmacy and Diabetes, School of Pharmacy and Pharmaceutical Science, Ulster University, Coleraine, County Londonderry BT52 1SA UK; 2 School of Biomedical Sciences, University of Ulster, Coleraine, Northern Ireland BT52 1SA UK

**Keywords:** Inflammation, Nanoparticles, Colitis, Hypoxia, Nuclear factor-kappa beta, Albumin

## Abstract

Hypoxia inducible factor and nuclear factor-kappa beta pathways have been proposed as therapeutic targets for several inflammatory diseases. Caffeic acid phenethyl ester (CAPE) and piceatannol (PIC) are natural anti-inflammatory compounds; however, poor bioavailability and limited understanding of biomolecular mechanistic limits its clinical use. The aims of this study are to enhance bioavailability and investigate their impact on nuclear p65 and HIF-1α for the first time in experimental colitis.

Dextran sulphate sodium was used to induce colitis in mice and effect of either free CAPE/PIC or CAPE/PIC loaded albumin nanoparticles treatment was observed on disease development and levels of cellular p65 and HIF-1α.

Our results indicate that albumin nano-encapsulation of CAPE/PIC not only enhances its anti-inflammatory potential but also potentiates its ability to effectively modulate inflammation related biomolecular pathways. Hence, combining nanotechnology with natural compounds could result in development of new therapeutic options for IBD.

**Electronic supplementary material:**

The online version of this article (10.1007/s13346-018-00597-9) contains supplementary material, which is available to authorized users.

## Background

Inflammation of the gastrointestinal tract is in fact a protective mechanism via which the host tries to protect itself from invading pathogens and noxious stimuli. The primary purpose of the inflammatory response is to remove and or inactivate the harmful substance; this process is supported by an array of cell-derived proteases and reactive oxygen products, as well as soluble mediators. Inflammation is normally self-limiting. However, in some cases, inflammation can become chronic leading to development of inflammatory bowel disease (IBD), a term commonly used to describe both ulcerative colitis (UC) and Crohn’s disease (CD). These gastrointestinal disorders have no effective treatment [1–3] and share common symptoms, such as weight loss, diarrhoea, rectal bleeding and abdominal cramp [4]. However, both disorders differ in affected location, with UC prevalent in the large intestine [5] and CD being of more variable occurrence in the gastrointestinal tract [6]. Both diseases present cryptic abscess, cryptitis and alteration in the mucosal layer [6]. The exact aetiology of IBD is unclear, but aberrant immunity, environmental factors, genetic disorders and dysregulation of the mucosal layer are thought to be responsible for the colitis [7]. These factors produce mucosal hypoxia and cause over activation of hypoxia-inducible factor 1 (HIF-1α) and nuclear factor-kappa beta (NF-kβ) [8, 9]. As a consequence, elevated levels of both transcription factors are frequently documented in colitis of the human colon [10].

UC occurs more frequently than Crohn’s disease [11] and damage to the mucosal layer is thought to be the main cause [12, 13]. The healthcare cost associated with management is considerable, especially in the USA, Europe and Asian countries [14–18]. The predominant drug-based therapies used in UC management, such as aminosalicylic acid, corticosteroids, 6-mercaptopurine, azathioprine and cyclosporine give rise to a range of debilitating side effects at various sites, such as skin, eyes and muscle [19, 20]. These therapeutic agents also induce bone marrow depletion and disorders in the lungs, liver and pancreas [21–23]. Additionally, these treatments are not only non-selective, but also impair lifestyle due to repeated use for protracted periods of time [24, 25]. Hence, simple drug-based interventions are of limited effectiveness in treating UC, leading inevitably to more series outcomes. These include elective procedures used to prevent further complications, such as colorectal cancer [26]. More sophisticated approaches based on novel and potent therapeutic agents, such as monoclonal antibodies, protease inhibitors and anti-TNF-α compounds, are also used in UC [27, 28] but their use has limitations due to side effects linked to liver damage, heart disease, infection, skin disease and degenerative disorders [25]. Arguably, the use of biomolecular drug substances offer few advantages, yet inflict toxicity, non-selectivity and a cost burden on patients [21].

There is a current and pressing need for alternative drug-based therapies to treat UC, which provide better healing, increase specificity and lower toxicity profiles, together with improved cost and convenience to patients. Compounds of natural origin, such as curcumin, caffeic acid phenethyl ester (CAPE) and piceatannol (PIC) could be used as adjutants to current clinically used anti-inflammatory, immune-suppressants and biologicals to improve therapeutic outcome, in the near future [22, 29]. PIC is naturally occurring agent present in grapes, red wine and passion fruit [30] and CAPE is derived from honeybee hives [31]. Together, they possess antioxidant properties and stabilise free radicals. They control inflammation and inhibit over stimulation of NF-kB and HIF-1α [32, 33]. They modulate overexpress of NF-kB by inhibiting pro-inflammatory mediators in colitis [34, 35] and prevent the binding of NF-kβ with DNA or inhibition of Ikβ in the cytoplasm [36, 37].

Although PIC and CAPE possess no known side effects and have beneficial activity that could be used to treat IBD, these compounds possess low solubility and poor bioavailability that limit their efficacy [38, 39]. Therefore, an aim of this work is to use nano-encapsulation to improve PIC and CAPE. The matrix selected was albumin, which is widely used as a biocompatible carrier that stabilises plasma pH, alleviates osmotic pressure and had been used to deliver proteins to cells [40]. Albumin nanoparticles (NP) have been shown to taken by cells due to inflammation at the mucosal site [41, 42]. Additionally, albumin is known to aggregate at sites of inflammation and tumour due to its affinity towards neutrophils, hence albumin nanoparticles could be used to target inflamed colon tissue in colitis [43].

There have been no previous published reports of albumin NP, loaded with PIC and CAPE, being evaluated in a murine model of chemically induced colitis. Our previous work has shown that CAPE is protective in experimental colitis at a dose of 30 mg kg^−1^ day^−1^ and was found ineffective at any lower doses [23]. Hence, in the present work, we have evaluated the effect of albumin NP loaded with PIC and CAPE at 20 mg kg^−1^ day^−1^ in a mouse (C57BL/6) model of DSS-induced colitis. The DSS model of colitis has been the first choice for colitis research since it is a simple model which produces robust results, however it lacks the immunological element of the disease; despite this shortcoming, it is known to produce an inflammatory response which is pathologically similar to human colitis [44].

The overarching aim of this study is to understand the dynamics of hypoxia inducible factor (HIF) and nuclear factor-kappa beta (NF-kβ) during active inflammation. Since, PIC and CAPE have limited solubility in aqueous media, we used a desolvation method to formulate albumin NP, loaded with PIC and CAPE to improve the solubility of these compounds. We have performed immuno-histochemistry and ELISA assays on colon tissue samples to quantify the activity of HIF-1a and nuclear p65 during active inflammation in experimental colitis.

## Methods

### Materials

Piceatannol (cat no.: P1928, purity > 98%, CAS: 10083-24-6) was obtained from Tokyo Chemical Industry UK Ltd., Oxford, UK. Caffeic acid phenethyl ester (cat no.: C8221, purity > 97%), albumin from bovine serum (cat no.: A2153, lot no.: 049k1585) and glutaraldehyde solution (cat no.: G5882) were obtained from Sigma Aldrich Ltd., Poole, UK. Dextran sodium sulphate (DSS) (cat no.: 160110, lot no.: M2356, molecular weight: 36000–50,000) was obtained from Fisher Scientific Ltd., Leicester, UK. Primary and secondary antibodies were purchased from Abcam, UK. All solvents used in the study were of HPLC grade and reagents were of analytical grade or higher. Ultrapure water was of type 1 standard (ISO 3696, Milli-Q®) and used throughout the study.

### Preparation of PIC/CAPE-loaded albumin nanoparticles

Albumin NP were loaded with either PIC or CAPE and fabricated using a desolvation method, with slight modification [45]. Briefly, CAPE/PIC (10 mg, 20 mg or 30 mg) was dissolved in ethanol (8.0 ml) and added to a 2% *w*/*v* BSA solution (2.0 ml) dropwise under constant stirring, leading to coacervate formation. An identical procedure using 10 mg, 20 mg or 30 mg of CAPE/PIC was used to prepare PIC-loaded NP. Coacervates were cross-linked using 8.0% *w/v* glutaraldehyde (100 μl) and stirred overnight at 500 rpm to remove traces of organic solvent. NP were collected by centrifugation (10,000×*g*) at 4 °C and washed three times with ultra-purified water. After washing, the albumin NP pellet was frozen at − 20 °C for 6 h and lyophilised (4.5 Plus, Labconco Ltd., USA) for 48 h.

### Albumin NP characterisation

#### Particles size and zeta potential

The particle size and distribution of PIC/CAPE-loaded albumin NP were measured using dynamic light scattering (Zetasizer 5000, Malvern Instruments, Malvern, UK). An aliquot of NP suspension (5 mg/ml) (1:5 dilution), previously vortex mixed, was diluted with ultrapure water and used to measure mean size and polydispersity index. Electrophoretic mobility was used to evaluate the zeta potential of PIC/CAPE-loaded albumin NP. Conductivity was adjusted using 0.001 M KCl. The average of three measurements was documented.

#### Entrapment efficiency

Entrapment efficiency was calculated using an indirect method. The concentration of non-encapsulated PIC/CAPE in the supernatant were measured by absorption spectrophotometry (CAPE at 425 nm/PIC at 325 nm) and compared to a standard plot. The amount of entrapped PIC/CAPE inside the NP was measured from the difference between the initial amount of PIC/CAPE added and the non-encapsulated PIC/CAPE remaining in the external aqueous phase after NP formulation. All measurements were recorded in triplicate and the average of each sample was expressed as the percentage of PIC/CAPE entrapment efficiency.

### Administration of PIC and CAPE-loaded albumin NP in mice

Five/six mice were assigned to groups, comprising healthy control, DSS control, free drug (PIC or CAPE) with DSS and PIC or CAPE-loaded albumin NP with DSS. Intraperitoneal injection of 20 mg/kg of free drug (PIC or CAPE) and equivalent amount (PIC or CAPE) of NP was administered in sterile PBS to mice.

### Induction of colitis and colon assessment

All animal procedures described in this work were approved by the Ulster University Animal Research Ethics Committee and UK Home Office, under project licence (PL2768). Colitis was induced in 12-week-old C57BL/six female mice (Charles River Ltd., Canterbury, UK) by administering 2.5% *w*/*v* DSS in drinking water over a period of 6 days. The disease activity index (DAI) score was used to record morphological changes, such as weight loss, stool consistency and presence of blood in the faeces. Upon termination of the experiment, mice were sacrificed by cervical dislocation [46]. The isolated colon was excised, washed in PBS and laid flat on moist tissue paper to measure its length. After removing the colonic content from each colon, its weight was measured to assess the change in colon weight. Approximately 1.0 cm of excised colonic tissue were fixed in 10% paraformaldehyde (pH 7.4; phosphate-buffered saline) and embedded in paraffin. Sections (4 μm) were cut and stained with haematoxylin and eosin. Histological assessment and scoring of colon tissue sections were carried out in a blinded fashion based on defined parameters, such as stool frequency, rectal bleeding, mucosal appearance and disease activity rating [47]. All tissue slides were imaged using optical microscope (AxioCam MRc ZEISS) light microscopy.

### Colon myeloperoxidases and cytokine measurements

The colonic tissue lysate was blended and homogenised as described by [23]. The expression of pro-inflammatory cytokines INF-γ, IL-6 and TNF-α, were quantified using V-Plex Assay Plates (Meso Scale Diagnostics; Rockville, MD, USA) and analyse as per the manufacturer’s. MPO activity was detected using *o*-phenylenediamine dihydrochloride as substrate and the data were interpolated from an MPO standard curve (Sigma). Expression of cytokines and MPO was represented as per milligram or U per milligram, respectively, relative to colonic protein.

### Immunohistochemistry

Colon tissues were stored in formalin and embedded in paraffin. Sections (5 μm) were mounted on glass slides (Superfrost Plus®), washed twice in xylene (10 min each) and rehydrated in decreasing concentration of ethanol for 5 min. It was then submerged in a glass jar filled with sodium citrate buffer (pH 6) kept in water bath at 95 °C, for 20 min. After heating for 20 min, the glass jar was removed from the water bath and allowed to cool at room temperature for 20 min. The slide was then washed in PBS twice for 5 min and blocking performed in 5% *w*/*v* BSA solution for 1 h at room temperature. Slides were washed twice again for 5 min and then incubated with secondary antibody for 1 h at room temperature. The tissue section was then stained with DAPI for 15 min and antifade was applied and sealed with coverslip. The sectioned was observed under an optical microscope (AxioCam MRc ZEISS) [48].

### Quantification of p65 and HIF-1α levels

The colonic tissue lysate from healthy, DSS, free drug of PIC or CAPE and PIC or CAPE-loaded albumin NP-treated mice were prepared and levels of p65 and HIF-1α were analysed using Invitrogen NF-kβ p65 (total) Human ELISA Kit (KHO0371) and HIF-1α Human ELISA Kit (EHIF1A) as per manufactures protocol at 450 nm using a BioTek optical plate reader. Optical density was converted to concentration (pg/ml) using the standard calibration curve provided in the manufactures protocol [49].

### Statistical analysis

Results were expressed as mean ± standard error of the mean (SEM) for a series of experiments. Data were assumed to be normally distributed and statistical analyses were carried out using Prizm GraphPad V6 software (GraphPad, San Diego, CA, USA). A paired *t* test was used for comparisons of paired treatments between two groups, unpaired *t* tests for comparisons of unpaired treatments between two groups and one-way ANOVA using Bonferroni multiple comparisons tests for treatments of three groups or more. *p* values ≤ 0.05 were considered to be significant [23].

## Results

### Optimisation of PIC and CAPE-loaded albumin NP

**Fig. 1:**
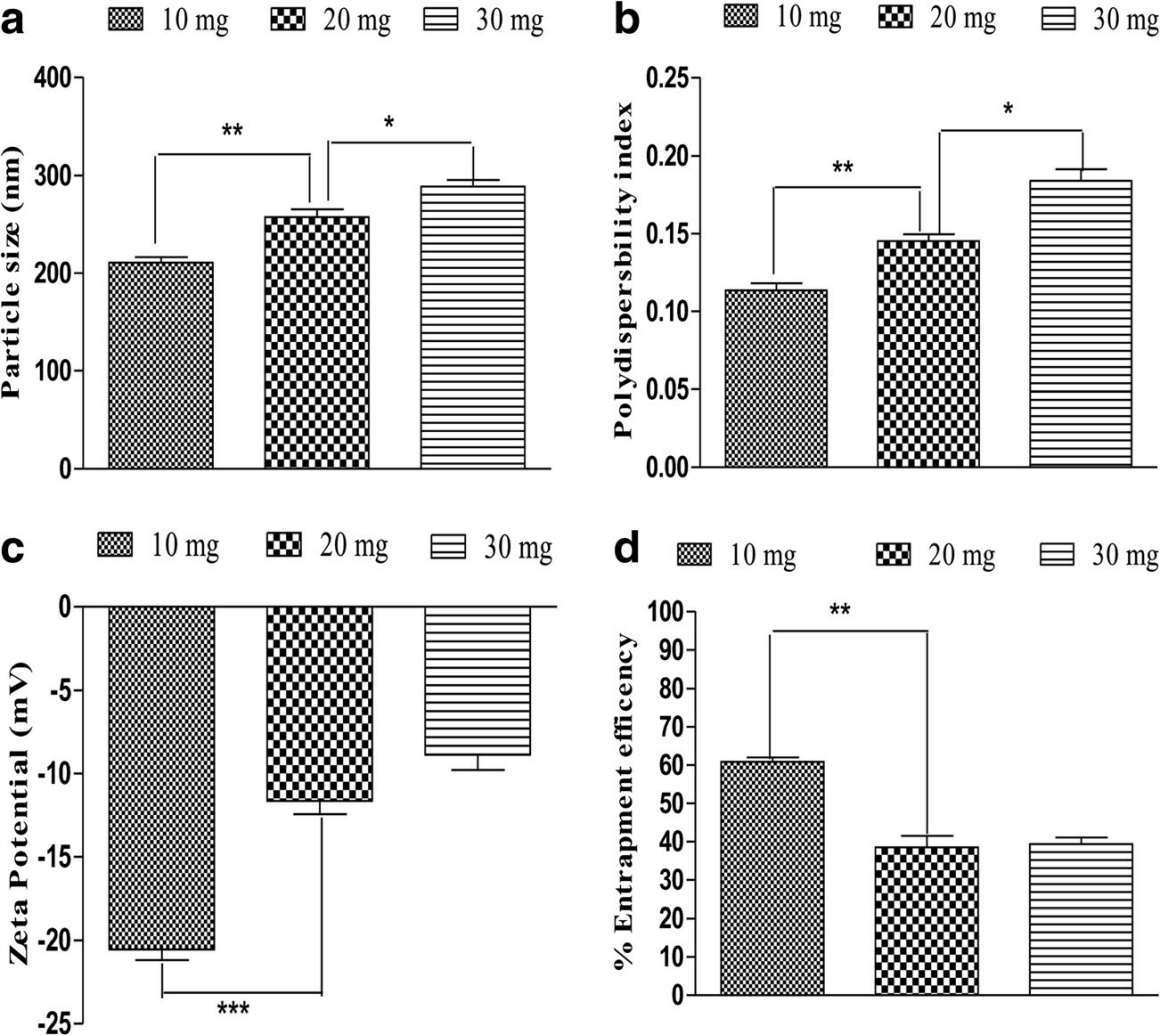
Effect of PIC loading on particle size, polydispersity index (PDI), zeta potential and entrapment efficiency. Increases in PIC concentration significantly (*p* < 0.01) increase the particle size (**a**), PDI (**b**) and (*p* < 0.001) zeta potential (**c**). Entrapment efficiency decreases significantly (*p* < 0.01) due to increase in PIC amount. Values are mean ± SEM (*n* = 3). *p < 0.05, **p < 0.01 and ***p < 0.001

**Fig. 2:**
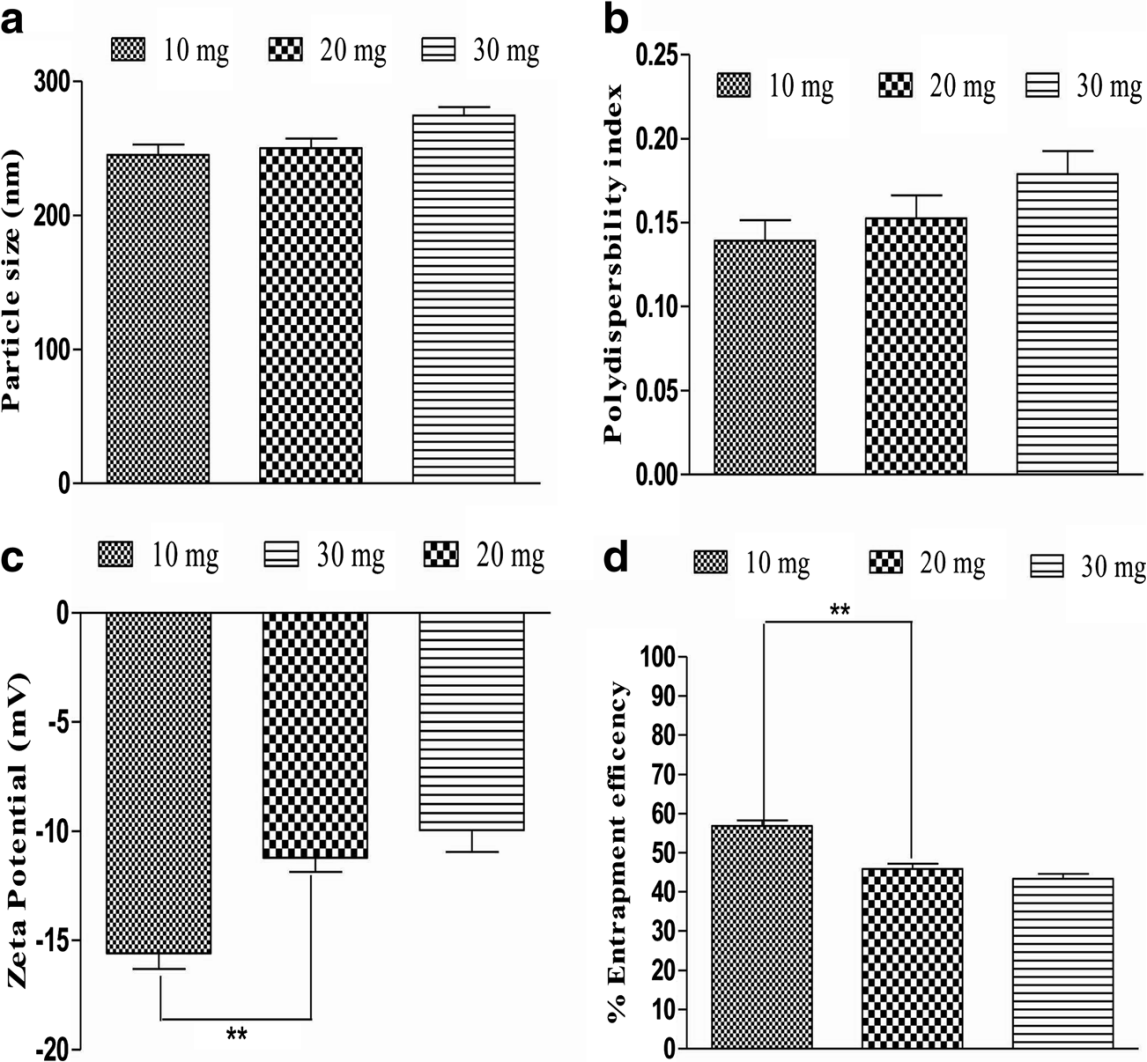
Effect of increase in CAPE concentration on particle size, polydispersity index (PDI), zeta potential and entrapment efficiency. Increases in CAPE concentration results in small (ns) increase the particle size (**a**) and PDI (**b**). However, zeta potential (**c**) increases significantly (*p* < 0.01) as CAPE amount increases. Entrapment efficiency (**d**) decreases significantly (*p* < 0.01) due to increase in CAPE amount. Values are mean ± SEM (*n* = 3). **p < 0.01

The effect of drug content was studied on particle size, polydispersity index and zeta potential. Drug-loaded albumin NP were formulated with 10 mg, 20 mg and 30 mg of free drug. An increase in particle size (210 to 288 nm) was observed when PIC amount increases from 10 to 30 mg (Fig. 1a). This increase in particle size may be due to increase amount of drug which in turn increases the viscosity of drug solution. Polydispersity index significantly (*p* < 0.01) increases (0.11 to 0.18) as drug amount increases (Fig. 1b). Likewise, zeta potential significantly (*p* < 0.001) increases (− 20 to − 8 mV) with increased PIC amount in the formulation (Fig. 1c). Additionally, entrapment efficiency significantly decreases (60 to 38%) due to increase in PIC concentration (Fig. 1d). However, increase in CAPE amount does not show any significant effect on particle size and polydispersity index as depicted in Fig. 2a, b. Although, significant (*p* < 0.01) increase in zeta potential (− 15 to − 9 mV) and decrease in entrapment efficiency (56 to 45%) was observed as illustrated in Fig. 2c, d.

### PIC and CAPE-loaded albumin NP alleviate weight loss and improves DAI in DSS-induced colitis

Mice were assigned to six groups, comprising healthy control, DSS 2.5% *w*/*v* alone, free drug (PIC/CAPE) along with DSS 2.5% *w/v* and PIC/CAPE-loaded albumin NP with DSS 2.5% *w/v*. Intraperitoneal injection of 20 mg/kg of free drug (PIC/CAPE) and equivalent amount of albumin NP containing CAPE/PIC was administered in sterile PBS to the mice. Change in weight was monitored daily and symptoms of colitis such as diarrhoea, weight loss and blood in faeces were reported as composite score of DAI. Post-mortem colon weight and length were noted on the final day [44, 50].

**Fig. 3:**
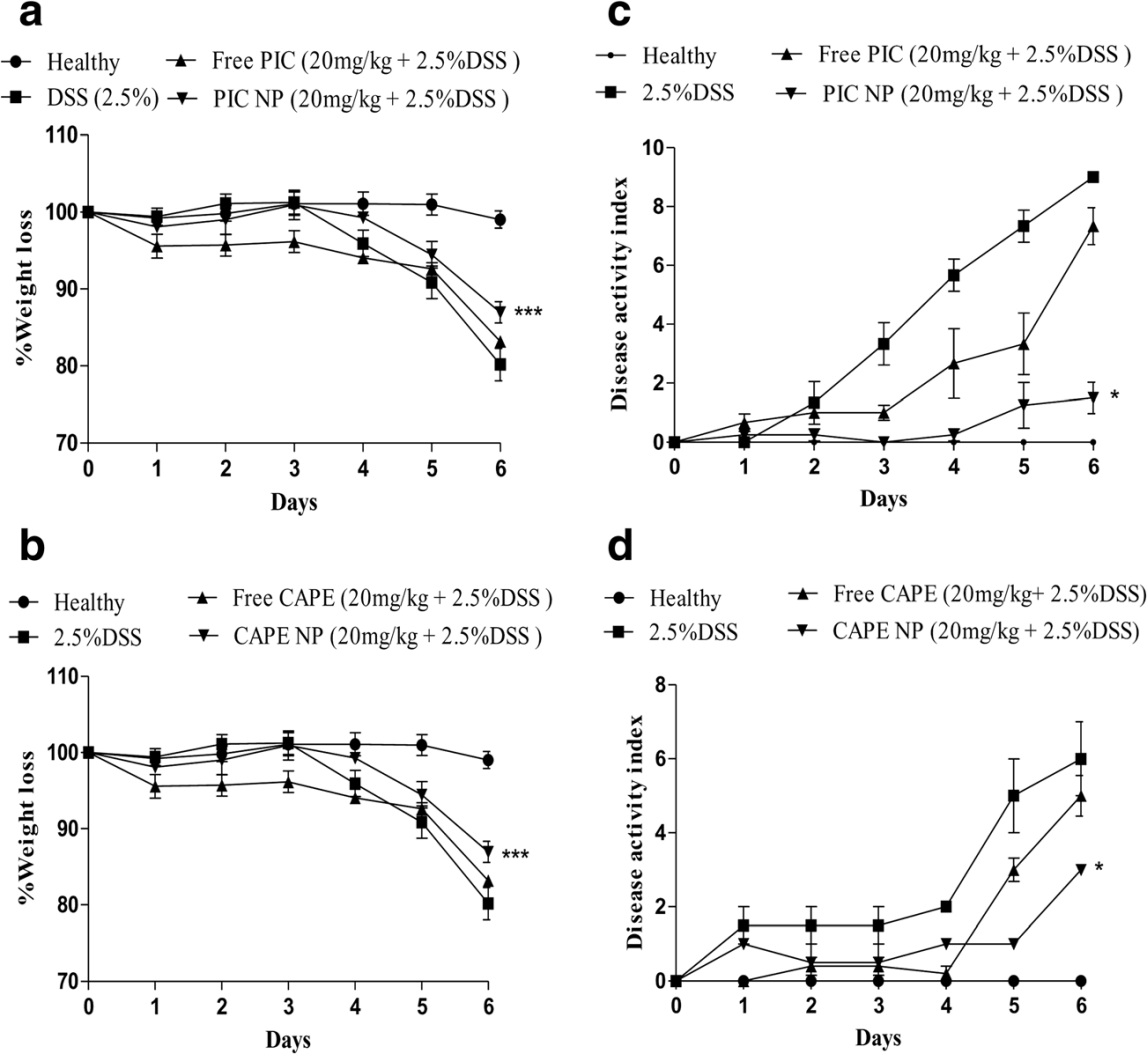
Effect of PIC and CAPE-loaded albumin NP treatment on percentage weight change and disease activity index (DAI). PIC (**a** and **c**) and CAPE (**b** and **d**)-loaded albumin nanoparticle treated group shows lower weight loss and low DAI as compared to the free PIC and CAPE-treated mice. *N* = 5–6 mice per group. *p < 0.05 and ***p < 0.001

Comparative protective activity of NP (PIC/CAPE) and free drugs (PIC/CAPE) was studied on C57BL/6 mice for 6 days. Documentation of weight of each mouse in all group were carried out daily. PIC/CAPE-loaded albumin NP + DSS group reported significantly (*p* < 0.001) less weight loss as compared to free PIC + DSS, free CAPE + DSS and DSS alone groups (Fig. 3a, b.

Furthermore, PIC/CAPE-loaded albumin NP + DSS group exhibit significant (*p* < 0.05) lower DAI score as compared to free PIC + DSS, free CAPE + DSS and DSS alone groups Fig. 3c, d.

The above finding suggests that PIC/CAPE-loaded albumin NP can significantly attenuate the inflammatory symptoms in experimental colitis such as diarrhoea, weight loss and blood in faeces as compared with free PIC/CAPE, indicating that albumin NP-based drug delivery can improve the therapeutic outcome.

### Colon morphology is improved after treatment with PIC/CAPE-loaded albumin NP in experimental colitis

**Fig. 4:**
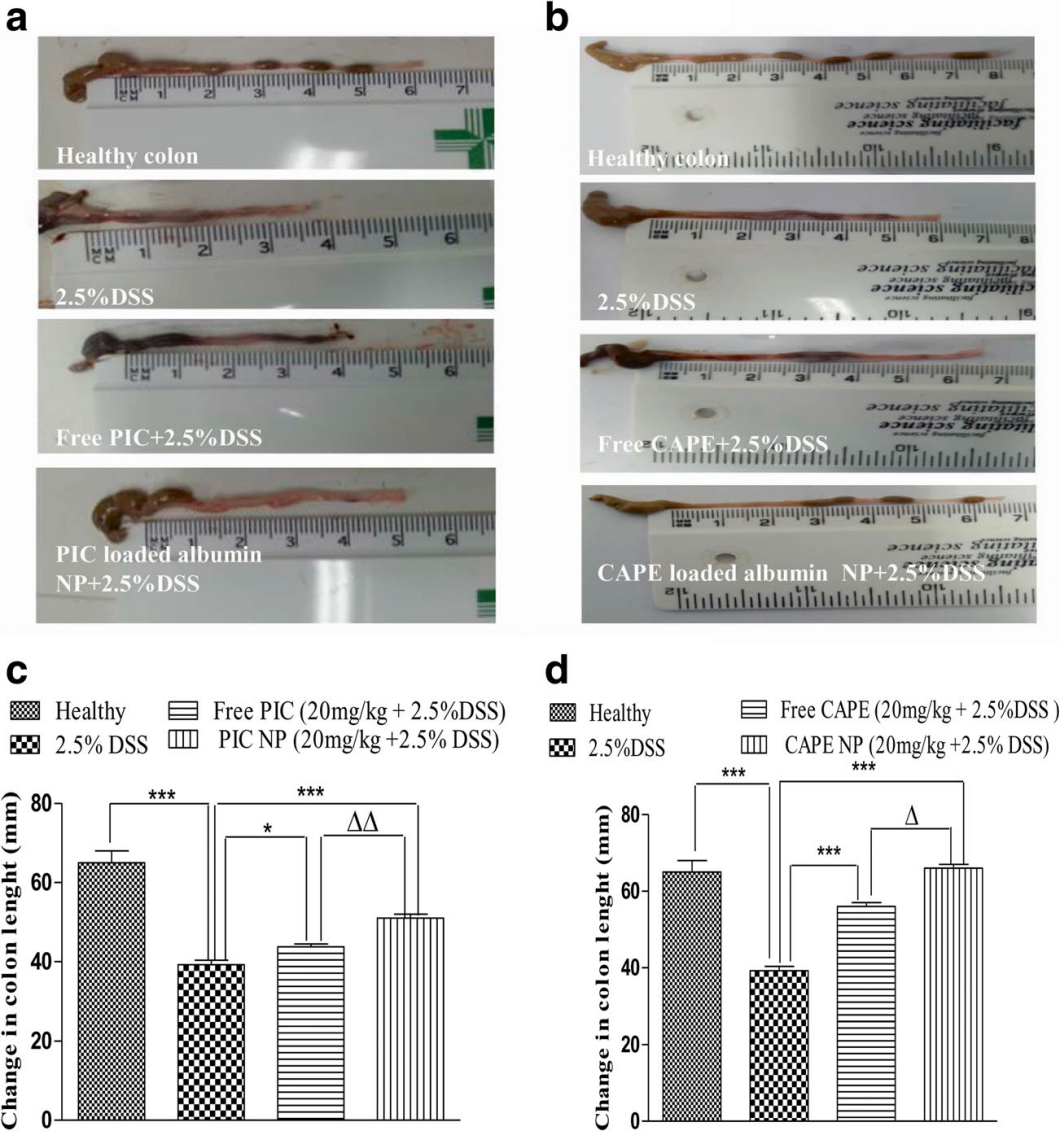
Effect of PIC and CAPE-loaded albumin NP treatment on colon morphology, length and weight. PIC/CAPE-loaded albumin NP + DSS treatment is effective in protecting gross anatomy and colon length as compared to the DSS group and free PIC/CAPE treatment. Gross appearance of the colonic anatomy shows the effect of PIC and CAPE-loaded albumin NP + DSS, free PIC and CAPE and DSS alone groups on colon shortening and formation of faecal pellets (**a**). The colon of mice treated with PIC nanoparticles closely resembles colon of the healthy group. However, free PIC shows loose stool, blood in faeces and shorter colon length same as the DSS treated group. Similarly, colon length and weight of PIC/CAPE-loaded albumin NP treated mice is not adversely effected (**b** and **c**). *N* = 5–6 mice per group. *p < 0.05 and ***p < 0.001, Δ*p* < 0.05 and ΔΔ*p* < 0.01

Shortening of colon length and alteration in colon morphology is other characteristics of human colitis and DSS-induced colitis in mice [51]. In our current study, effect of free drugs (PIC/CAPE) and albumin NP containing PIC/CAPE on post-mortem colon length and weight was recorded (Fig. 4). The stool in the healthy group appeared to be normal (Fig. 4 A first picture). Loose stool with a blood clot is seen in the colon of mice treated with DSS only and free PIC/CAPE along with DSS, (Fig. 4a). PIC-loaded albumin NP (Fig. 4a, picture 4) and CAPE-loaded albumin NP (Fig. 4a, picture 4) shows no sign of blood clots during experimental colitis. Well-formed stool pellets can be seen in CAPE-loaded albumin NP (Fig. 4a, picture 4).

PIC/CAPE-loaded albumin NP illustrates significant (*p* < 0.01/*p* < 0.05) higher colon length than DSS alone and free PIC/CAPE groups (Fig. 4b). Treatment of PIC-loaded albumin NP (*p* < 0.05) and CAPE-loaded albumin NP (*p* < 0.05) in mice protects the loss in colon weight (Fig. 4c) as compared to free PIC and CAPE and DSS alone groups. Therefore, above data suggest that PIC/CAPE-loaded albumin NP improves the overall morphology of the colon during active colitis in a murine model of DSS-induced colitis.

### Histological investigation in mice treated with PIC/CAPE-loaded albumin NP

**Fig. 5:**
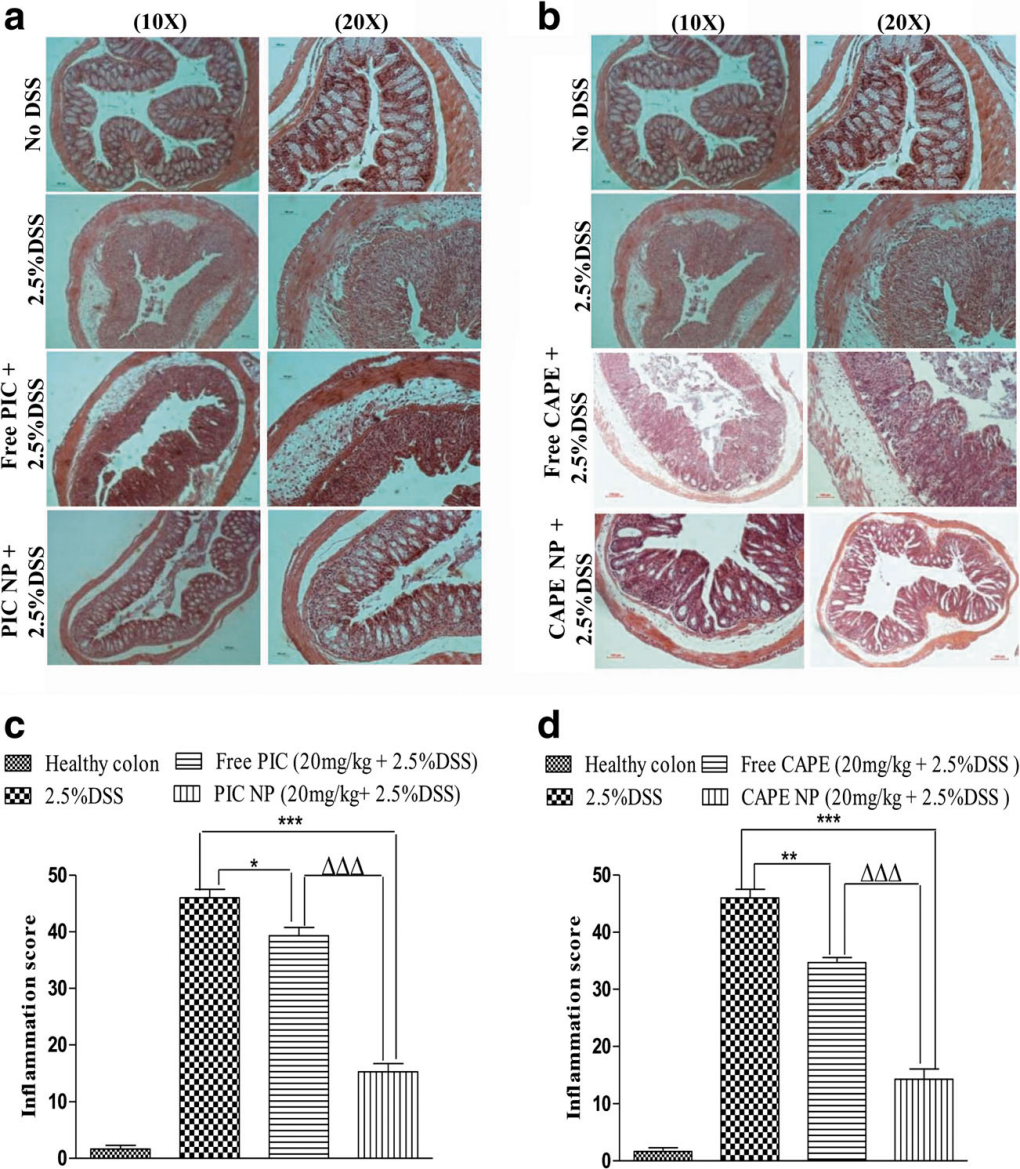
Effect of PIC and CAPE-loaded albumin NP treatment on colon histology and inflammation score. Improved colon histological outcome in mice treated with PIC and CAPE-loaded albumin NP + DSS (**a**). The cryptic structure in the epithelial layer of healthy PIC and CAPE-loaded albumin NP group is identical (**a**). However, infiltration of cytokines and the distorted epithelial layer can be observed in the free PIC and CAPE-treated mice (**a**). Significant (*p* < 0.0001) low inflammation was recorded in PIC/CAPE-loaded albumin NP-treated group is reported as compared to free PIC/CAPE groups (**b**). *N* = 5–6 mice per group. *p < 0.05, **p < 0.01 and ***p < 0.001. ΔΔΔ*p* < 0.001

Histological investigation of colonic epithelia treated with DSS demonstrated an alteration in structural integrity along with permeation of inflammatory neutrophils inside the distorted epithelial layer in mouse colon of DSS alone and free PIC/CAPE-treated groups (Fig. 5a). However, the degree of cryptic epithelial damage and infiltration of inflammatory cells is very low in PIC/CAPE-loaded albumin NP-treated mice as compared to free PIC/CAPE and DSS groups (Fig. 5a). Blinded tissue inflammation scores of colonic tissues histology indicates significant (*p* < 0.001) reduction of inflammation score (composite score of crypt damage and inflammation) in PIC/CAPE-loaded albumin NP-treated mice relative to DSS control and free PIC/CAPE-treated mice (Fig. 5b).

### Colon myeloperoxidases and cytokine measurements

**Fig. 6:**
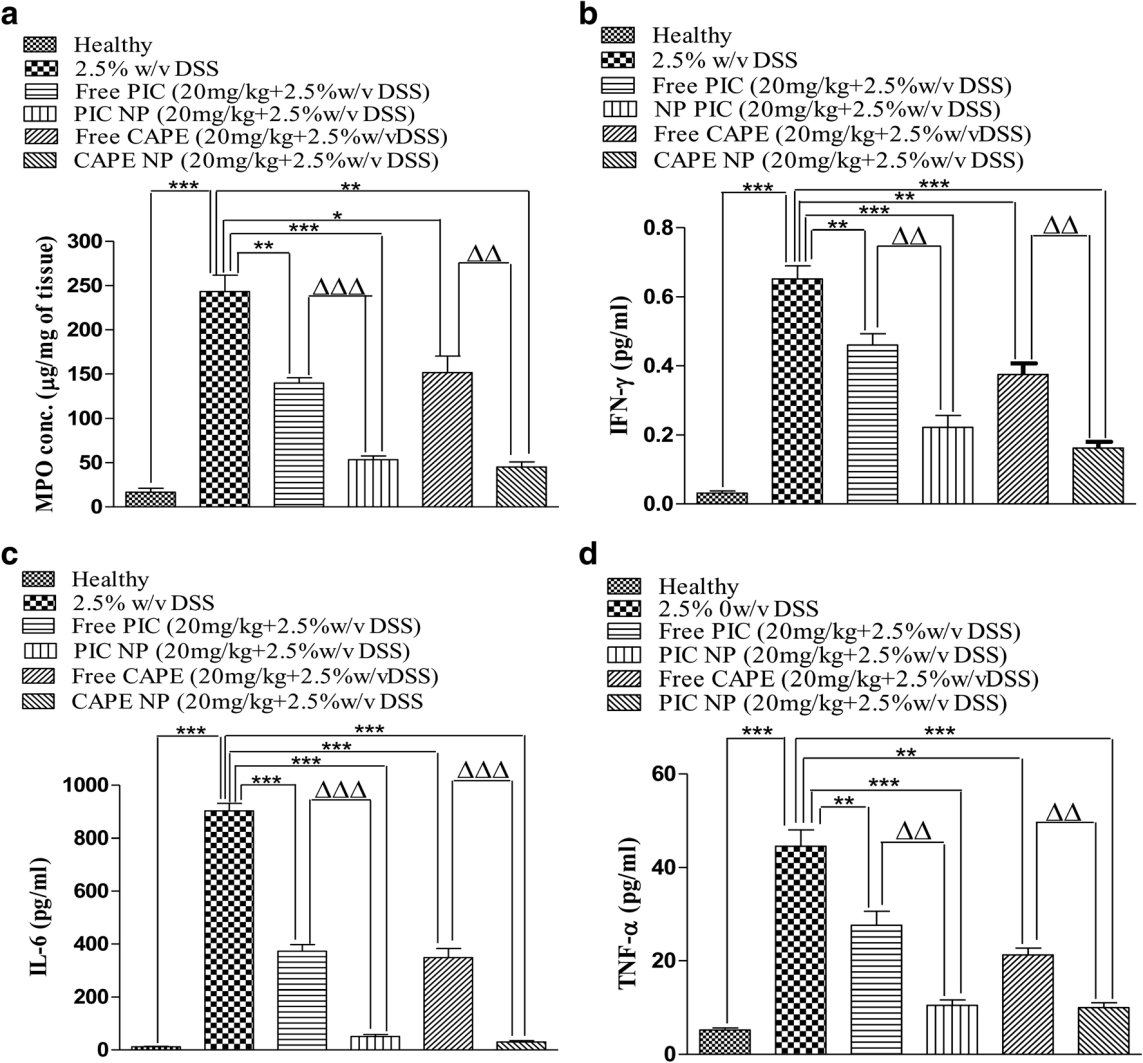
Effect of free PIC/CAPE and PIC/CAPE-loaded albumin NP on expression of pro-inflammatory mediators. The colon tissue homogenates analysed for MPO (**a**), INF-ϒ (**b**), IL-6 (**c**) and TNF-α (**d**). It was noted that PIC/CAPE-loaded albumin decreases the expression of inflammatory markers when compared with free PIC/CAPE. *p < 0.05, *p* < 0.01 and ***p < 0.001 compared with the healthy group. ΔΔ*p* < 0.01 and ΔΔΔ*p* < 0.001 compared with the same dose of free PIC/CAPE molecules. *N* = 5–6 mice per group

We further examined the expression of colonic inflammatory markers in mice colon tissue when exposed to DSS and being treated with free PIC/CAPE and albumin-loaded PIC/CAPE. DSS alone control mice showed a significant increase in MPO levels, a marker for inflammation and leukocyte infiltration (Fig. 6a). There was attenuation of MPO expression in mice treated with free PIC/CAPE; however, mice treated with PIC/CAPE-loaded albumin NP reported significant (*p* < 0.01) reduction of MPO as compared to DSS alone and free PIC/CAPE groups (Fig. 6a). Similarly, the level of inflammatory markers such as INF-γ, IL-6 and TNF-α decreases significantly (*p* < 0.01) in PIC/CAPE-loaded albumin NP-treated groups as compared with DSS alone and free PIC/CAPE groups (Fig. 6b–d).

### Level of p65 and HIF-1α in PIC and CAPE-loaded albumin NP tissue

**Fig. 7:**
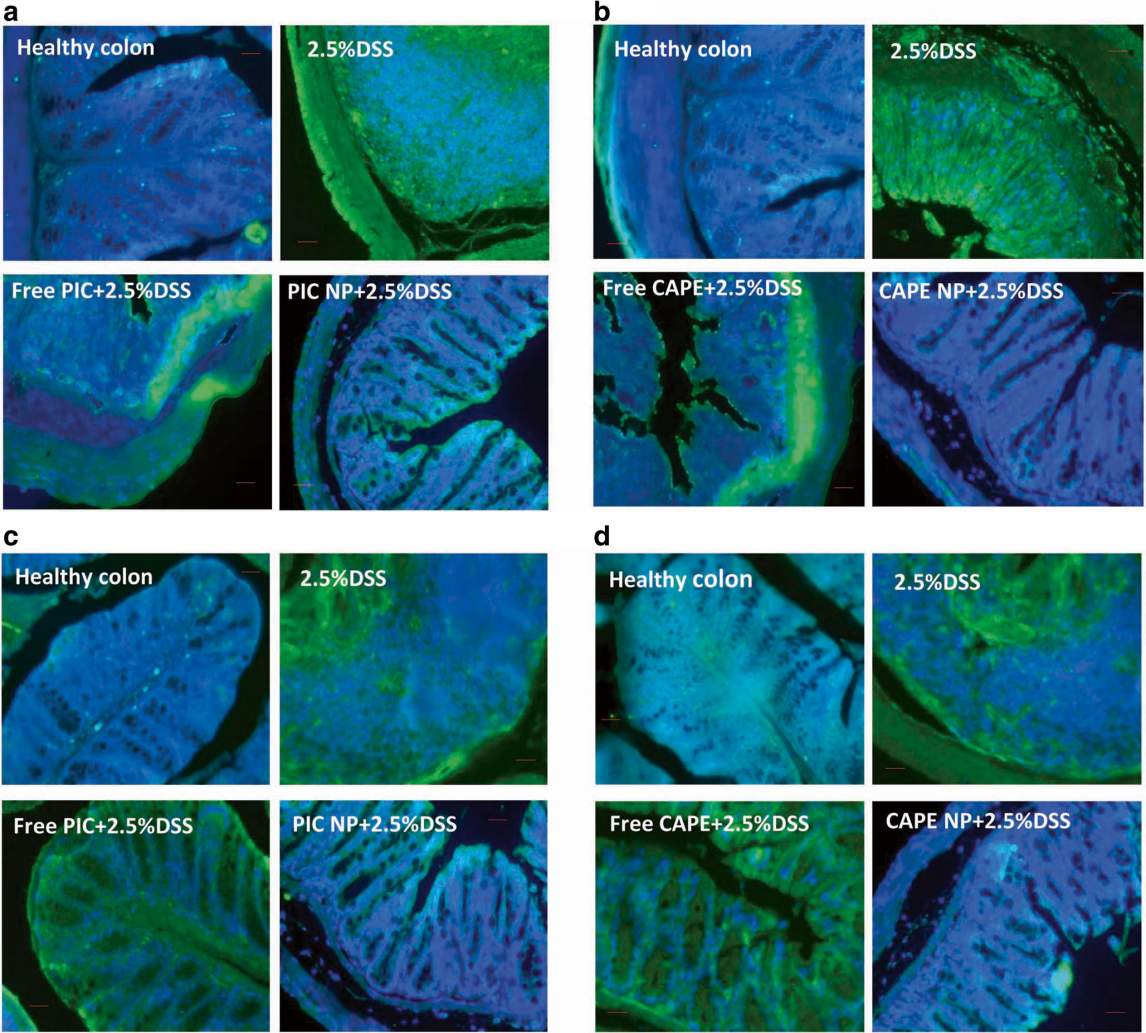
Effect of PIC and CAPE-loaded albumin NP on transcription proteins p65 and HIF-1α in colon. Lower levels of p65 in PIC and CAPE-loaded albumin NP + DSS-treated group is observed by immuno-histochemistry (**a**) and ELISA assay (**b**). Similarly, expression of HIF-1α decreases significantly in PIC and CAPE-loaded albumin NP-treated mouse colon when assessed by immuno-histochemistry (**c**) and ELISA assay (**d**). Values are mean ± SEM. *p < 0.05, *p* < 0.01 and ***p < 0.001 compared with the healthy group. ΔΔΔ*p* < 0.001 compared with the same dose of free PIC/CAPE molecules. *N* = 5–6 mice per group

Expression of HIF and NF-kβ transcription proteins namely HIF-1α and p65 was observed in mouse colon tissue using fluorescence immunohistochemistry. Noticeable decrease in the expression of HIF-1α and p65 was observed in colonic tissue of mice treated with PIC/CAPE-loaded albumin NP as compared to mice treated with free molecule of PIC/CAPE; colon tissues of mice exposed to 2.5% DSS show higher expression of both HIF-1α and p65 (Fig. 7a, c).

Furthermore, level of p65 and HIF-1α in colonic tissue lysate was assessed using ELISA assay (Supplementary Fig. 1). It was observed that PIC/CAPE-loaded albumin NP significantly (*p* < 0.001) decreased the expression of p65 and HIF-1α as compared to free molecules of PIC/CAPE (Fig. 7b, d).

## Discussions

Therapeutic activity of PIC and CAPE against several disorders is supported by the presence of their phenolic nature [52]. However, it has been documented that phenolic compounds are poorly soluble in aqueous media which limit their efficacy [53]. Hence, we have formulated PIC and CAPE as albumin nanoparticles to overcome solubility and bioavailability issues. Our previous work indicates that CAPE was protective in experimental colitis via reduction in levels of pro-inflammatory mediators and enhancement of epithelial barrier function [23]. Our current work indicates that enhanced therapeutic outcome can be achieved at a lower dose when CAPE and PIC is delivered as albumin nanoparticles.

In the present study, we have tested albumin-loaded nanoparticles containing PIC and CAPE for the first time in a DDS-induced murine model of colitis. Effect of drug amount of PIC and CAPE on particle size, polydispersity index, zeta potential and entrapment efficiency were studied. It was found that increase in drug concentration leads to increase in particle size, polydispersity index and zeta potential (Figs. 1 and 2). However, entrapment efficiency decreases due to increase in drug amount. Thus, after careful optimisation, we selected 20-mg drug concentration to formulate albumin NP loaded with PIC and CAPE for evaluation of the anti-inflammatory potential of PIC and CAPE in chemically induced mouse model of colitis. Parameters such as DAI, rectal bleeding, shortening of colon length, weight of colon and alteration of epithelial layer of colon in DSS induce colitis were also accessed during evaluation of anti-inflammatory activity in vivo [54–56]. Early signs of colon inflammation such as weight loss and increase DAI score were reported in DSS alone group as shown in Fig. 3, this indicates that the presence of DSS in drinking water induces colitis in mice. However, these symptoms were absent in PIC and CAPE-loaded albumin NP. Free PIC and CAPE-treated mice showed some degree of attenuations these symptoms during active colitis (Fig. 3). These findings indicate that PIC and CAPE can exert anti-inflammatory effect during active colitis which is potentiated when these compounds are formulated as albumin nanoparticles.

After dissection of the mice and isolation of the colon gross morphology was observed appreance of blood clots, loose stool and shortening of colon was reported in DSS alone, free PIC and CAPE-treated group (Fig. 4a). PIC and CAPE-loaded albumin NP-treated mice exhibit normal stool, no blood and the equivalent colon length compared with the healthy group as depicted in Fig. 4a. Furthermore, no significant difference in colon length PIC/CAPE albumin NP-treated groups was observed when compared healthy group (Fig. 4b). The entire colon weight was recorded, colons from of PIC and CAPE-loaded albumin NP-treated mice illustrate no significant difference in weight as compared with healthy groups, a small degree of loss in colon weight was reported in free PIC and CAPE-treated mice (Fig. 4c). These findings indicate that administration of PIC and CAPE as albumin nanoparticles is highly effective in protecting the gross morphology of the colon during active colitis.

Treatment with PIC and CAPE-loaded albumin NP resulted in marked improvement in colon histology (Fig. 5a), together with improved blinded inflammation scores (Fig. 5b). In murine model of DSS-induced colitis, the increase in MPO and pro-inflammatory cytokines occurred after the onset of colon inflammation due to physical barrier disruption. PIC and CAPE-loaded albumin NP-treated mice show no increase in MPO (Fig. 6a) and only small increase in other pro-inflammatory cytokines (Fig. 6b–d).

Several previous findings indicate inflammation results in overstimulation of transcription protein such as p65 and HIF-1α in ulcerative colitis and colorectal cancer which results in damage of epithelial layer of colon [57, 58]. Overactivation of nuclear factor p65 and HIF-1α has also been reported in UC patients [59, 60]. Colon tissue from mice treated with DSS alone showed upregulation of both nuclear factor p65 and HIF-1α during active inflammation as compared with healthy mice (Fig. 7a–d). Treatment with free CAPE and PIC results in small reduction of p65 and HIF-1α; this effect is significantly enhanced when CAPE and PIC is administered as albumin nanoparticles (Fig. 7a–d). Hence, we can conclude that the therapeutic anti-inflammatory potential of CAPE and PIC to modulate the hypoxia inducible factor and nuclear factor-kappa beta pathways can be improved by using an inflammation targeting drug delivery system (Supplementary Figure 1). Thus nanotechnology could play an important role in improving therapeutic potential of natural compounds such as CAPE and PIC resulting in the development of a novel therapeutic option for inflammatory bowel diseases.

## Electronic supplementary material

ESM 1 (PNG 210 kb)
