# Supplementary figures and images for: Albumin nano-encapsulation of caffeic acid phenethyl ester and piceatannol potentiated its ability to modulate HIF and NF-kB pathways and improves therapeutic outcome in experimental colitis
Source: Drug Deliv Transl Res. 2018 Nov 14;9(1):14–24. doi: 10.1007/s13346-018-00597-9 (PMC6328632; doi:10.1007/s13346-018-00597-9)

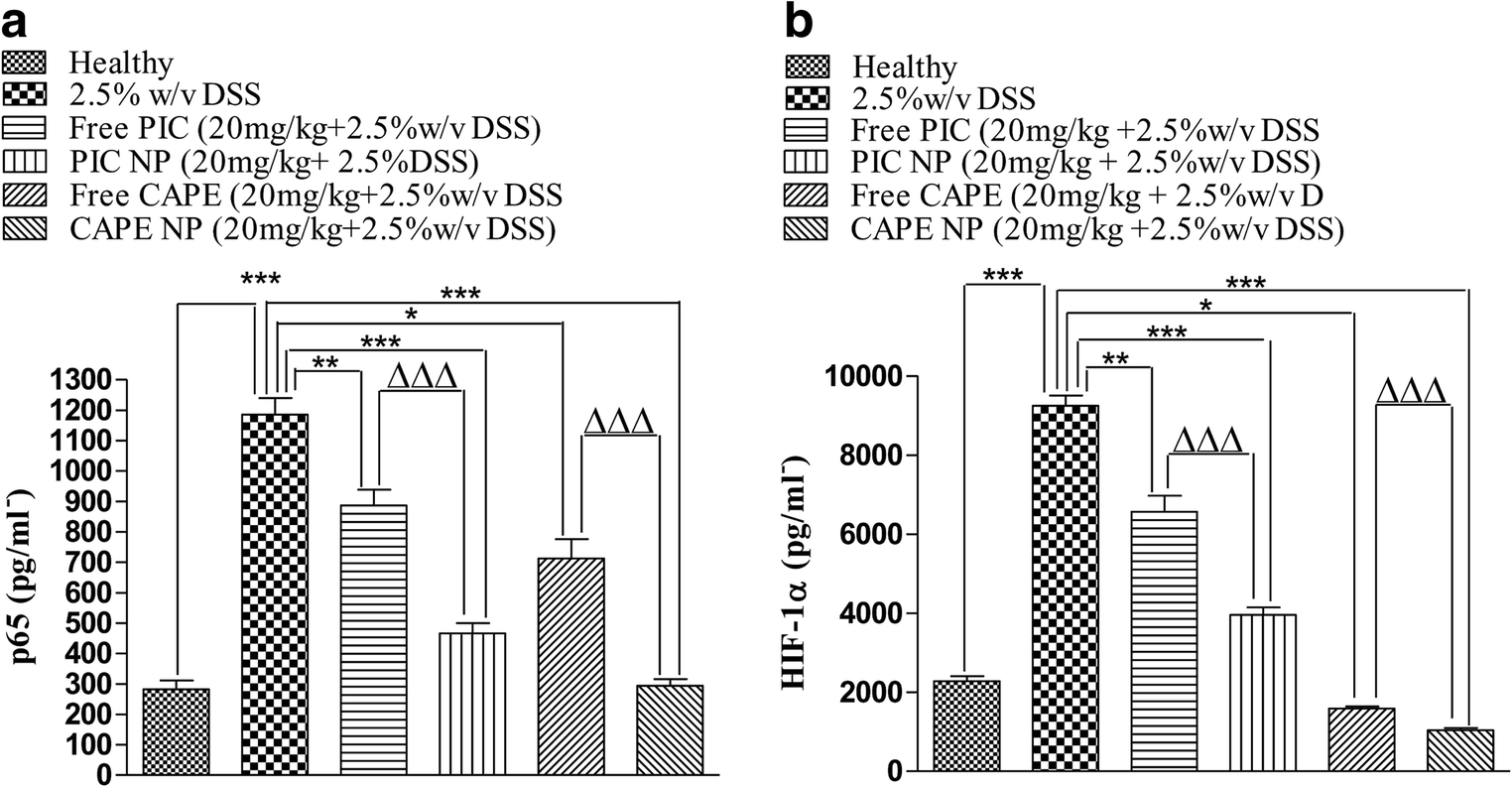

Supplement: Supplementary file 1 — (PNG 210 kb) [file 13346_2018_597_MOESM1_ESM.png]
